# Exhausted Tumor-infiltrating CD39^+^CD103^+^ CD8^+^ T Cells Unveil Potential for Increased Survival in Human Pancreatic Cancer

**DOI:** 10.1158/2767-9764.CRC-23-0405

**Published:** 2024-02-19

**Authors:** Laia Gorchs, Carlos Fernández-Moro, Ebba Asplund, Marlies Oosthoek, Martin Solders, Poya Ghorbani, Ernesto Sparrelid, Elena Rangelova, Matthias J. Löhr, Helen Kaipe

**Affiliations:** 1 Department of Laboratory Medicine, Karolinska Institutet, Stockholm, Sweden.; 2 Department of Pathology and Cancer Diagnostics, Karolinska University Hospital, Stockholm, Sweden.; 3 Department of Upper GI, C1:77 Karolinska Comprehensive Cancer Center, Stockholm, Sweden.; 4 Department of Clinical Science, Intervention and Technology, Karolinska Institutet, Stockholm, Sweden.; 5 Department of Surgery, Section for Upper Abdominal Surgery, Sahlgrenska University Hospital, Gothenburg, Sweden.; 6 Clinical Immunology and Transfusion Medicine, Karolinska University Hospital, Stockholm, Sweden.

## Abstract

In pancreatic ductal adenocarcinoma, the infiltration of CD8^+^ T cells within the tumor microenvironment correlates with a favorable prognosis. However, a significant proportion of tumor-infiltrating T cells become trapped within the desmoplastic stroma and lack tumor reactivity. Here, we explored different T-cell subsets in pancreatic tumors and adjacent tissues. We identified a subset of CD8^+^ T cells, double positive (DP) for CD39 and CD103 in pancreatic tumors, which has recently been described to display tumor reactivity in other types of solid tumors. Interestingly, DP CD8^+^ T cells preferentially accumulated in central tumor tissues compared with paired peripheral tumor and adjacent non-tumor tissues. Consistent with an antigen encounter, DP CD8^+^ T cells demonstrated higher proliferative rates and displayed an exhausted phenotype, characterized by elevated expression of PD-1 and TIM-3, compared with CD39^−^CD103^−^ CD8^+^ T cells. In addition, DP CD8^+^ T cells exhibited higher expression levels of the tissue trafficking receptors CCR5 and CXCR6, while displaying lower levels of CXCR3 and CXCR4. Importantly, a high proportion of DP CD8^+^ T cells is associated with increased patient survival. These findings suggest that DP CD8^+^ T cells with a phenotype reminiscent of that of tumor-reactive T cells are present in pancreatic tumors. The abundance of DP CD8^+^ T cells could potentially aid in selecting patients for pancreatic cancer immunotherapy trials.

**Significance:**

Patients with pancreatic cancer with a high proportion of CD39^+^CD103^+^ CD8^+^ T cells exhibiting a tumor-reactive phenotype have improved survival rates, suggesting their potential utility in selecting candidates for immunotherapy trials.

## Introduction

Pancreatic cancer has a 5-year overall survival (OS) rate of less than 10% and its prognosis has not improved significantly over the last three decades (1). While the infiltration of CD8^+^ T cells in pancreatic ductal adenocarcinomas (PDAC) has been associated with an improved OS (2–4), current immunotherapies are ineffective (5). This ineffectiveness might be attributed to the spatial distribution of T cells, with the majority confined in the distinctive abundant desmoplastic stroma and excluded from the tumor nests (4, 6–10). Of note, one study has demonstrated that CD8^+^ T-cell infiltration in close proximity to cancer cells is associated with improved survival (4). This suggests that the fibrotic reaction might not always hinder T-cell infiltration, and that there might be an active antitumor immune response.

Chemokine receptors can provide crucial signals to the T cells to promote an effective tumor immunosurveillance. For instance, CXCR3 and CCR5 have been shown to be involved in T-cell trafficking into the tumors (11–13), whereas CXCR6 is involved in providing survival signals to T cells in the tumor microenvironment (14, 15), and CXCR5 in the migration of T cells into tertiary lymphoid structures (TLS; refs. 16, 17). In contrast, the CXCL12/CXCR4 axis is involved in the retention of T cells in the tumor stroma of pancreatic cancer (18).

The αE-integrin CD103 binds to the epithelial cell marker E-cadherin and identifies tissue-resident memory CD8^+^ T cells (19). CD8^+^ CD103^+^ T cells are associated with a favorable survival prognosis in several cancers, such as lung cancer and urothelial cell carcinoma (20–22). CD39 is an ectonucleotidase, which together with CD73, is involved in converting ATP into immunosuppressive adenosine (23). T cells upregulate CD39 upon persistent antigen exposure and is hence associated to exhausted T cells with high expression of inhibitory receptors (24, 25). Recently, the coexpression of CD39 and CD103 on CD8^+^ T cells has been reported to be associated with tumor-reactive T cells in various types of solid cancers, including head and neck squamous cell carcinoma and ovarian cancer (26, 27). A subset of CD4^+^ T cells expressing CD39 has also been shown to exhibit tumor reactivity (28). An increasing number of single-cell RNA sequencing studies have thoroughly examined the immune landscape in PDAC (6, 10, 29, 30). These studies provide evidence of antigen processing and immune activation (29), along with the presence of putatively tumor-reactive CD39^+^ CD103^+^ CD8^+^ T cells (30). However, the relevance and phenotype of this putatively tumor-reactive T-cell subset as well as the location in PDAC remains unknown.

In the current study, we have investigated the phenotype of T cells from the central and peripheral parts of the tumor, as well as adjacent nonaffected tissues of resected pancreatic tumor tissues, to improve our understanding of T-cell subsets infiltrating tumor-rich areas. We demonstrated that CD8^+^ T cells coexpressing CD39 and CD103 accumulate in the central parts of the tumor, present an exhausted phenotype with high expression of immune checkpoint markers, and display a distinct chemokine receptor expression pattern. A high proportion of CD39^+^CD103^+^ CD8^+^ T cells is associated with increased patient survival, suggesting tumor reactivity.

## Materials and Methods

### Patient Samples

A total of 59 patients with resectable pancreatic cancer provided written informed consent and were enrolled in the study (Table 1). The patients underwent surgery at the Upper Gastrointestinal Disease Unit of Karolinska University Hospital, Huddinge, Sweden between 2018 and 2022. Peripheral blood was collected from the patients before surgery. The study was conducted in accordance with the Declaration of Helsinki and the experimental protocols were approved by the Regional Review Board of Ethics in Research in Stockholm (entry nos. 2018/1792-31/2, 2019/0-3500).

**TABLE 1:** Patient characteristics

Variables	*n* = 59
Demographic characteristic	
Female gender, *n* (%) Median age, years (range)	29 (49) 69 (50–88)
Male gender, *n* (%) Median age, years (range)	30 (51) 69 (51–82)
Oncologic characteristics	
Histologic type, *n* (%)	
Pancreatic ductal adenocarcinoma	57 (97)
Adenosquamous carcinoma	2 (3)
Tumor depth, *n* (%)	
T2	12 (20)
T3	44 (75)
T4	3 (5)
Lymph node metastasis, *n* (%)	
N0	7 (12)
N1	41 (69)
N2	11 (19)
Metastasis, *n* (%)	
M0	52 (88)
M1	7 (12)
Preoperative chemotherapy, *n* (%)	
Yes	11 (19)
No	48 (81)

NOTE: T2, Tumor >2 cm; T3, Tumor > 4 cm; T4, Tumor involves coeliac axis, superior mesenteric artery and/or common hepatic artery; N0, No regional lymph node metastasis; N1, Metastases in one to three regional lymph nodes; N2, Metastases in four or more regional lymph nodes; M0, No distant metastasis; M1, Distant metastasis.

### Tissues and Blood Collection

Subspecialists in pancreatic pathology at the Pathology Unit at Karolinska University Hospital in Huddinge divided the resected tissues into three different areas: a central part of the tumor [mean weight 160 mg (range: 50–300 mg)], a peripheral part of the tumor [mean weight 169 mg (range: 40–430 mg)], and a non-tumorous adjacent tissue [mean weight 209 mg (range: 50–490 mg)]. The sampling technique is shown in Supplementary Fig. S1A. The tissues were placed in DMEM culture medium (Cytiva, catalog no. SH30021.01) supplemented with 10% FBS (Cytiva, catalog no. SV30160.03) and 1% PEST (Cytiva, catalog no. SV30010) and processed immediately for cell isolation. A reference piece from each tissue area was also collected by pathologists, fixed in 4% formalin, embedded in paraffin, and processed for histology and IHC as matched reference tissues. Peripheral blood was collected from all patients on the same day prior to the surgery.

### Cell Isolation

Tumor-infiltrating lymphocytes (TIL) were isolated from all three tissue areas by mechanical disaggregation, using a GentleMACS Tissue Dissociator (RRID:SCR_020267; Miltenyi Biotec). The cell suspension was filtered through a 70-µm cell strainer (VWR), washed, and either stained immediately for FACS analysis or cryopreserved until further analysis in RPMI1640 (Cytiva, catalog no. SH3035501), supplemented with 10% FBS and 1% PEST (complete RPMI) with 10% Dimethyl sulfoxide (DMSO) (WAK-Chemie catalog no. WAK-DMSO-70) until further analyses.

Peripheral blood mononuclear cells (PBMC) from peripheral blood from patients were isolated by a density gradient over Ficoll-Paque PLUS (Cytiva, catalog no. 17144003). PBMCs were either used fresh for FACS analysis or cryopreserved.

### Flow Cytometry

The TILs and PBMCs were stained with mAbs (Supplementary Table S1) in PBS supplemented with 2 mmol/L Ethylenediaminetetraacetic acid (EDTA) and 0.2% BSA for 20 minutes at 4°C. For extracellular staining, 7-Aminoactinomycin D (7AAD) was used to distinguish live cells from dead. For intracellular staining, the cells were fixed and permeabilized using a BD Cytofix/Cytoperm kit [Becton Dickinson (BD), catalog no. 554714] according to the manufacturer's instructions. Fixability viability staining was used to distinguish between the live and dead cells. Cells were acquired on a FACSCanto II (BD Biosciences) or CytoFlex (Beckman Coulter). The MR1-5-OP-RU tetramers technology for MAIT cell stainings was developed jointly by Dr. James McCluskey, Dr. Jamie Rossjohn, and Dr. David Fairlie, and the material was produced by the NIH Tetramer Core Facility as permitted to be distributed by the University of Melbourne (Melbourne, Australia; ref. 31). Data were analyzed using FlowJo (BD, version 10.7.2. RRID: SCR_008520) by manual gating, by high-dimensional single-cell data analysis using t-distributed stochastic neighbor embedding (t-SNE), or by clustering using a self-organizing map (FlowSOM).

### Quantification of T-cell Subsets

The total number of CD4^+^ and CD8^+^ T cells was counted using flow cytometry and divided by the collected tissue weight resulting in an estimated number of cells/mg of tissue. If more than one characterization panel was run, the median numbers of CD4^+^ and CD8^+^ T cells were used.

### IHC Stainings

Consecutive sections from the embedded tissues were analyzed by IHC analysis. A total of 4-µm-thick sections were immunohistochemically stained for CK19 (clone A53-B/A2.26, Cell Marque) and visualized with alkaline phosphatase AP chromogen (red) using a Leica BOND III automated immunostainer. The slides were digitalized using a Hamamatsu NanoZoomer S360 scanner (RRID: SCR_023761). Areas with desmoplastic stroma and tumor were manually annotated to calculate the proportions of affected areas versus the residual normal tissue out of the total tissue on whole slide images using QuPath (RRID: SCR_018257; ref. 32). The proportion of the tissue that contained the tumor was recorded from the stroma/tumor areas and adjusted for the total tissue area.

### Statistical Analysis

Wilcoxon matched pairs signed-rank test was used to detect differences between two paired groups. Friedman test, followed by Dunn multiple comparison test, was used to detect differences between three or more paired groups. OS was calculated from the time of surgery to the time of death or last follow-up and represented using Kaplan–Meier curves. The log-rank test was used for the statistical analyses. We compared the OS between patients with high and low expression levels of certain markers using the median as a cutoff. The hazard ratio (HR) for DP CD8^+^ T cells and clinicopathologic characteristics including age, sex, T, N, M stage, levels of CA 19-9 and neoadjuvant chemotherapy were defined by multivariate Cox proportional hazards regression. Statistical significance was set at *P* < 0.05. All statistical analyses were performed using GraphPad Prism version 9 (RRID: SCR_002798).

### Data Availability

The data generated in this study are available upon request from the corresponding authors.

## Results

### T Cells Infiltrate Both Tumor-rich Tissues and Non-tumor Tissues

**FIGURE 1:**
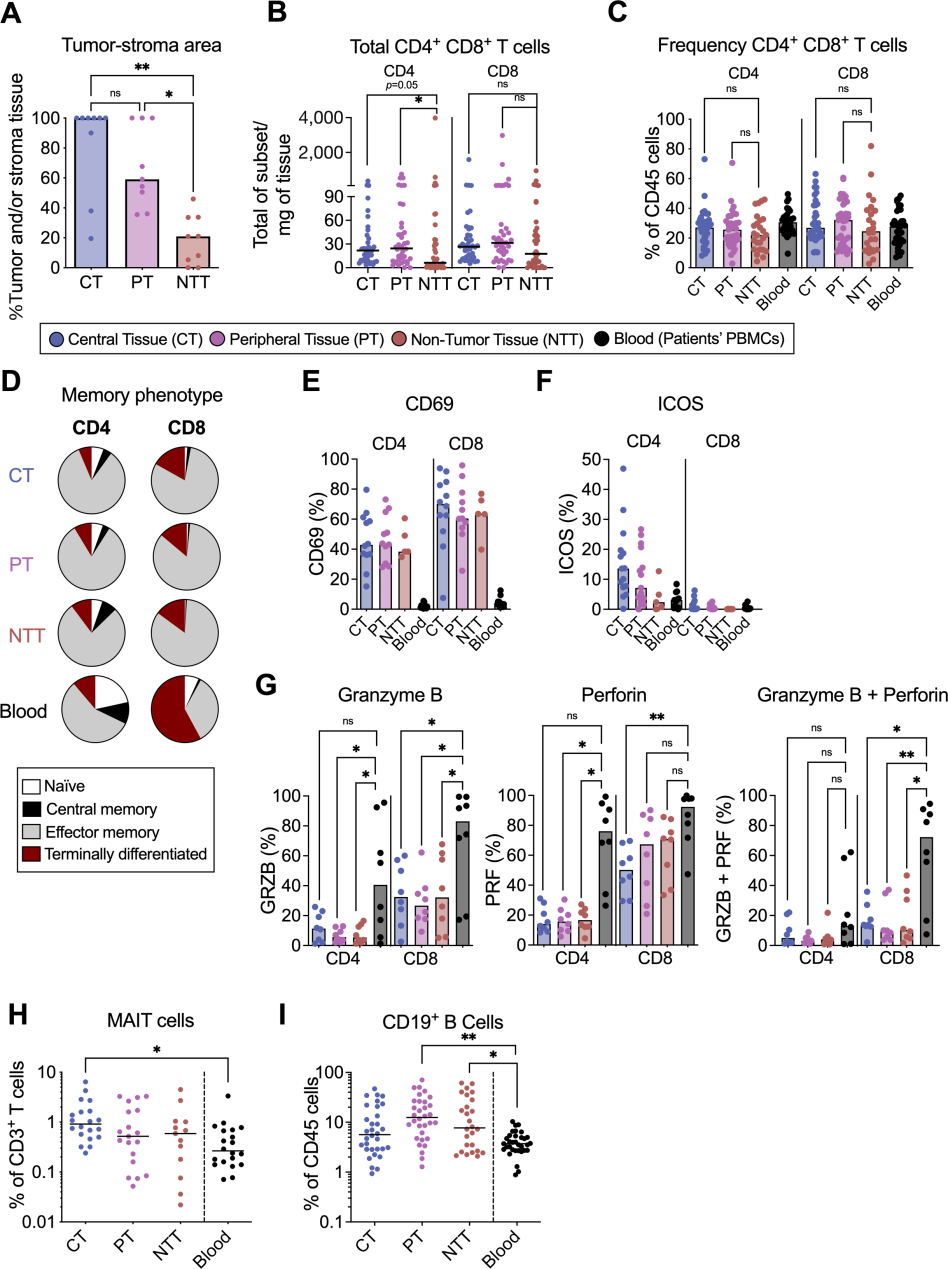
Proportion of immune cells in pancreatic tissues. The proportion of different immune subsets were studied by flow cytometry in central tissues (CT; blue), peripheral tissues (PT; purple), non-tumor tissues (NTT; brown), and patients’ PBMCs (black). **A,** Tumor and reactive stroma area calculated by QuPath in the three tissue compartments (*n* = 9). **B,** Total numbers of CD4^+^ and CD8^+^ T cells per mg of tissue (*n* = 40). **C,** Frequency of CD4^+^ and CD8^+^ T cells from CD45^+^ cells (CT, PT *n* = 35, NTT *n* = 25, blood *n* = 35). **D,** Memory phenotype of CD4^+^ and CD8^+^ T cells (CT *n* = 15, PT *n* = 14, NTT *n* = 6, blood *n* = 24). **E,** Proportion of CD69 on CD4^+^ and CD8^+^ T cells (CT *n* = 12, PT *n* = 11, NTT *n* = 5, blood *n* = 12). **F,** Proportion of ICOS on CD4^+^ and CD8^+^ T cells (CT *n* = 15, PT *n* = 14, NTT *n* = 5, blood *n* = 15). **G,** Proportion of granzyme B, perforin, and granzyme B-perforin coexpression on CD4^+^ and CD8^+^ T cells from central and peripheral tumor tissues, non-tumor tissues and patients’ PBMCs (*n* = 8). **H,** Proportion of MAIT cells from CD3^+^ T cells (CT *n* = 21, PT *n* = 20, NTT *n* = 13, blood *n* = 21). **I,** Proportion of CD19^+^ B cells from CD45^+^ cells (CT *n* = 32, PT *n* = 32, NTT *n* = 26, blood *n* = 32). **A–C, F–I,** Friedman test followed by Dunn test was used to evaluate significant difference between groups (CT, PT, NTT; F–I) and patients’ PBMCs if *n* > 5. *, *P* <0.05; **, *P* < 0.01; ***, *P* < 0.001; ns, not significant.

To obtain an overview of the tissue composition of the resected central, peripheral, and non-tumor tissues, the proportion of tissue consisting of tumor and desmoplastic stroma was manually annotated in 9 patients using QuPath (Supplementary Fig. S1B). Figure 1A shows that most of the central tissues consisted of tumor/desmoplastic stroma, with a median of 100%, while the peripheral tissues had a median of 57%. The non-tumor tissues (tissues adjacent to the tumors) were often affected by pancreatitis and fibrosis but had no detectable tumor cells (median 14% of the area). The remaining tissues consisted of normal residual pancreatic tissue or fat. Lymphocytes from the central, peripheral, and non-tumor areas of freshly resected pancreatic tumor tissues were isolated, and the frequencies and phenotypes of CD4^+^ and CD8^+^ T cells were studied by flow cytometry. The gating strategy for the main lymphocyte subsets is presented in Supplementary Fig. S1C.

The total number of CD4^+^ and CD8^+^ T cells within the tissues was determined by flow cytometry and normalized to tissue weight. CD4^+^ T cells were more prevalent in the central and peripheral tissues than in the non-tumor tissues (Fig. 1B). However, when analyzing the proportion of CD4^+^ and CD8^+^ cells out of CD45^+^ cells, we found no significant differences between the tissue compartments (Fig. 1C). The total number of CD4^+^ and CD8^+^ T cells in the tissues was not associated with survival in our patient cohort (Supplementary Fig. S2A).

T cells isolated from central, peripheral, and non-tumor tissues predominantly displayed an effector memory phenotype (CD45RA^−^ CCR7^−^; Fig. 1D). Moreover, a large proportion of T cells expressed CD69, which was uniformly expressed across tissues (Fig. 1E). ICOS was mainly expressed on CD4^+^ T cells, with large variability between patients (Fig. 1F). The median proportion of ICOS^+^ CD4^+^ T cells in central tissues was 4-fold higher than that in non-tumor tissues and peripheral blood T cells. Altogether, this suggests that pancreatic TILs present a memory and tissue residency phenotype, independent of their proximity to tumor cells.

To assess the cytotoxic potential of CD4^+^ and CD8^+^ pancreatic TILs, we measured the expression of the cytolytic molecules granzyme B and perforin in T cells isolated from both tumor and patient PBMCs (Fig. 1G). CD4^+^ T cells from peripheral and non-tumor tissues expressed lower levels of granzyme B and perforin than paired circulating CD4^+^ T cells (Fig. 1G). These differences were even more pronounced in CD8^+^ T cells, where CD8^+^ T cells in central, peripheral, and non-tumor tissues contained less granzyme B and coexpressed less granzyme B and perforin than blood CD8^+^ T cells (Fig. 1G).

The frequency of MAIT cells, which are an invariant type of cytotoxic T cells with an unknown function in cancer, was examined in pancreatic cancer tissues. MAIT cells were identified using the MR1 tetramers (Supplementary Fig. S1C). Even though no statistically significant differences were found between the three tissue compartments (Fig. 1H), the central tumor tissue contained a higher proportion of MAIT cells (1.05%) out of CD3^+^ T cells compared with paired peripheral blood (0.34%) (Fig. 1G), which could suggest that MAIT cells home to tumor-rich tissues. The MAIT cell proportions were not associated with increased patient survival (Supplementary Fig. S2B).

B cells in PDAC tumors are primarily found in the TLSs (33, 34). We examined the frequency of CD19^+^ B cells among CD45^+^ cells (Supplementary Fig. S1C) in tissues and blood and observed an accumulation of B cells in the peripheral and non-tumor tissues compared with the patient PBMCs (Fig. 1I). This suggests that B cells are homing to pancreatic tissues but are primarily localized to peritumoral areas. The proportion of B cells was not predictive of OS (Supplementary Fig. S2C).

### T Cells in Tumor-rich Tissues Display a More Exhausted Phenotype Compared with Tumor-free Regions

**FIGURE 2:**
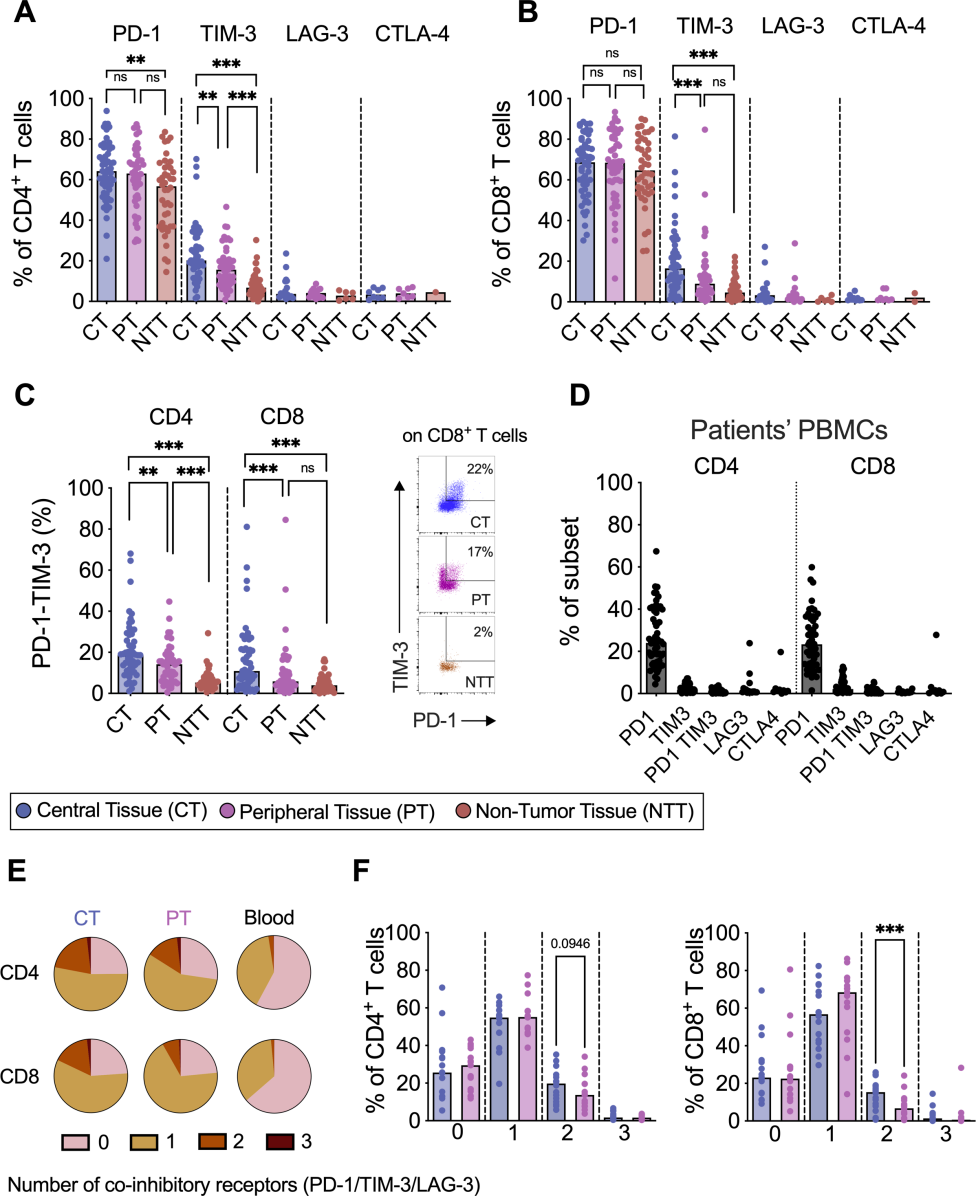
Coinhibitory receptor expression on CD4^+^ and CD8^+^ tumor-infiltrating T cells. Proportion of CD4^+^ (**A**) and CD8^+^ (**B**) T cells expressing PD-1 (CT *n* = 54, PT *n* = 52, NTT *n* = 42), TIM-3 (CT *n* = 53, PT *n* = 51, NTT *n* = 41), LAG-3 (NTT *n* = 6, CT *n* = 16, PT *n* = 16), CTLA-4 (CT *n* = 9, PT *n* = 8,NTT *n* = 2), and coexpressing PD-1 and TIM-3 coinhibitory markers (CT *n* = 53, PT *n* = 51, NTT *n* = 41; **C,** left), flow cytometry representative dot plots on CD8^+^ T cells (right) to identify coexpression of PD-1 and TIM-3. **D,** Proportion of PD-1, TIM-3, PD-1TIM-3, LAG-3, and CTLA-4 in CD4^+^ and CD8^+^ T cells from patients’ PBMCs. **E,** Boolean gating analysis was performed on TIM-3, PD-1, and LAG-3 (*n* = 16). Pies (left) show median values on central tumor tissues (CT), peripheral tumor tissues (PT), and patients’ PBMCs (blood). **F,** Proportion of CD4^+^ and CD8^+^ T cells expressing 0, 1, 2, or 3 coinhibitory markers in central and peripheral tumor tissues. **A–C,** Friedman test followed by Dunn test was used to evaluate significant difference between groups (CT, PT, NTT) if *n* > 5. **E,** Wilcoxon matched pairs signed-rank test was used to detect statistically significant differences between CT and PT tissues. *, *P* < 0.05; **, *P* < 0.01; ***, *P* < 0.001; ns, not significant.

Next, we examined the expression of coinhibitory markers in T cells from tumor-rich and non-tumor tissues. A large proportion of CD4^+^ and CD8^+^ T cells expressed PD-1 in all the three tissue compartments. CD4^+^ T cells in central tissues expressed more PD-1 than those in non-tumor tissues (Fig. 2A), but no significant differences were found in CD8^+^ T cells across the tissues (Fig. 2B). In contrast, TIM-3 expression was significantly higher in the central than in the peripheral and non-tumor tissues in both CD4^+^ and CD8^+^ T cells (Fig. 2A and B). A similar pattern was observed for the coexpression of PD-1 and TIM-3 in CD4^+^ and CD8^+^ T cells (Fig. 2C). A median of less than 10% of CD4^+^ and CD8^+^ T cells expressed LAG-3 and CTLA-4 across the tissue compartments, with no significant differences (Fig. 2A and B). CD4^+^ and CD8^+^ T cells in blood expressed low levels of TIM-3, LAG-3, and CTLA-4, and a median of 25% and 24% of PD-1, respectively (Fig. 2D). Together, this suggests that TIM-3 expression more accurately identifies T cells that have encountered malignant cells compared with PD-1, considering its more uniform expression across tissues.

Boolean gating was used to determine whether the T cells expressed none, one, two, or three of the coinhibitory markers PD-1, TIM-3, and LAG-3. Figure 2E shows that the majority of T cells in tissues expressed one or two of the coinhibitory receptors and rarely coexpressed all three, whereas the majority of peripheral blood T cells expressed none or one of the coinhibitory markers (Fig. 2E). Both CD4^+^ and CD8^+^ T cells in the central tissues coexpressed at least two coinhibitory receptors at a higher frequency than T cells in the peripheral tissues (Fig. 2F). The most commonly coexpressed receptors in central tissues were PD-1 and TIM-3, with a median of 20% for CD4^+^ T cells and 10% for CD8^+^ T cells (Fig. 2C), whereas the median of PD-1 and LAG-3 coexpression was below 5% (Supplementary Fig. S3). The non-tumor tissues contained too few events for Boolean gating and were, therefore, omitted from the analysis.

### The Expression of Chemokine Receptors CCR5 and CXCR6 on T Cells is Associated to an Exhausted Phenotype

**FIGURE 3:**
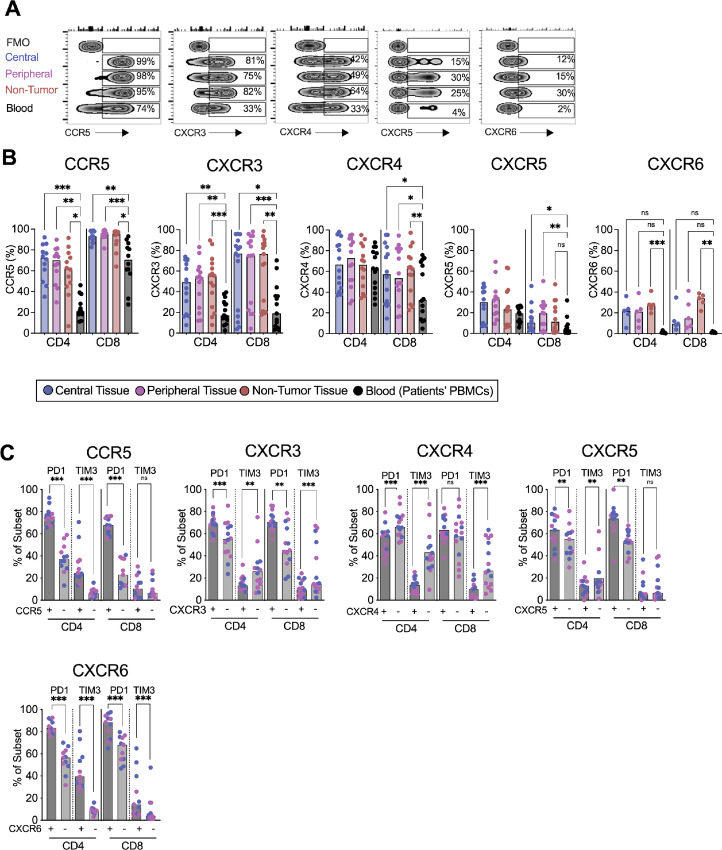
Expression of coinhibitory markers on T cells expressing chemokine receptors. **A,** Representative flow cytometry zebra plots on CD8^+^ T cells showing the gating strategy to identify CCR5, CXCR3, CXCR4, CXCR5, and CXCR6 positive cells. **B,** Proportion of CCR5 (*n* = 12), CXCR3 (*n* = 15), CXCR4 (*n* = 14), CXCR5 (*n* = 12), and CXCR6 (*n* = 6) on CD4^+^ and CD8^+^ T cells from central and peripheral tumor tissues, non-tumor tissues and patient PBMCs. **C,** Expression of PD-1, TIM-3, and PD-1-TIM-3 coexpression on CD4^+^ and CD8^+^ T cells expressing (+) or not (−) CCR5, CXCR3, CXCR4, CXCR5, and CXCR6 in central and peripheral tumor tissues of 6–7 patients. **B,** Friedman test followed by Dunn test was used to evaluate significant difference between groups. **C,** Wilcoxon matched pairs signed-rank test was used to detect statistically significant differences. *, *P* < 0.05; **, *P* < 0.01; ***, *P* < 0.001; ns, not significant.

The expression of chemokine receptors on TILs may have important implications for their migratory capacity toward malignant cells. Both CD4^+^ and CD8^+^ T cells showed a significantly higher expression of CCR5 and CXCR3 in tissues compared with circulating T cells (Fig. 3A and B), but they were both uniformly expressed in tumor-rich areas and non-tumor tissues. CXCR4 and CXCR5 were also more highly expressed in tumor-rich tissues than in circulating T cells, but only in CD8^+^ T cells (Fig. 3A and B). CD8^+^ CXCR5^+^ cells were significantly enriched in peripheral tumor tissues compared with circulating T cells (Fig. 3A and B). We further found that CXCR6 was expressed over 20-fold and 10-fold more in CD4^+^ and CD8^+^ T cells, respectively, in tissues than in circulating T cells. However, owing to the limited number of samples, only the expression of CXCR6 on T cells in non-tumor tissues was significantly higher than that in circulating T cells (Fig. 3A and B).

Next, we analyzed the expression of PD-1 and TIM-3 in T cells positive or negative for CCR5, CXCR3, CXCR4, CXCR5, and CXCR6 in central and peripheral pancreatic tissues (Fig. 3C). CCR5^+^ CXCR3^+^, CXCR5^+^, and CXCR6^+^ T cells displayed a higher expression of PD-1 than T cells not expressing these chemokine receptors. Furthermore, CCR5^+^ and CXCR6^+^ T cells expressed higher levels of TIM-3 than CCR5^−^ and CXCR6^−^ T cells. Interestingly, CXCR3^+^ and CXCR5^+^ CD4^+^ T cells presented lower levels of TIM-3 than CXCR3^−^ and CXCR5^−^ CD4^+^ T cells, respectively. In contrast, CXCR4^+^ T cells expressed lower levels of both PD-1 and TIM-3 than CXCR4^−^ T cells (Fig. 3C).

Altogether, T cells in tumor-rich areas did not show upregulation of chemokine receptors compared with T cells in non–tumor-rich areas. However, CD4^+^ and CD8^+^ T cells expressing CCR5 and CXCR6 and lacking CXCR3 and CXCR4 seem to have a more exhausted phenotype with an upregulation of TIM-3.

### CD39^+^ CD103^+^ T Cells are Present in Pancreatic Tumor Tissues

**FIGURE 4:**
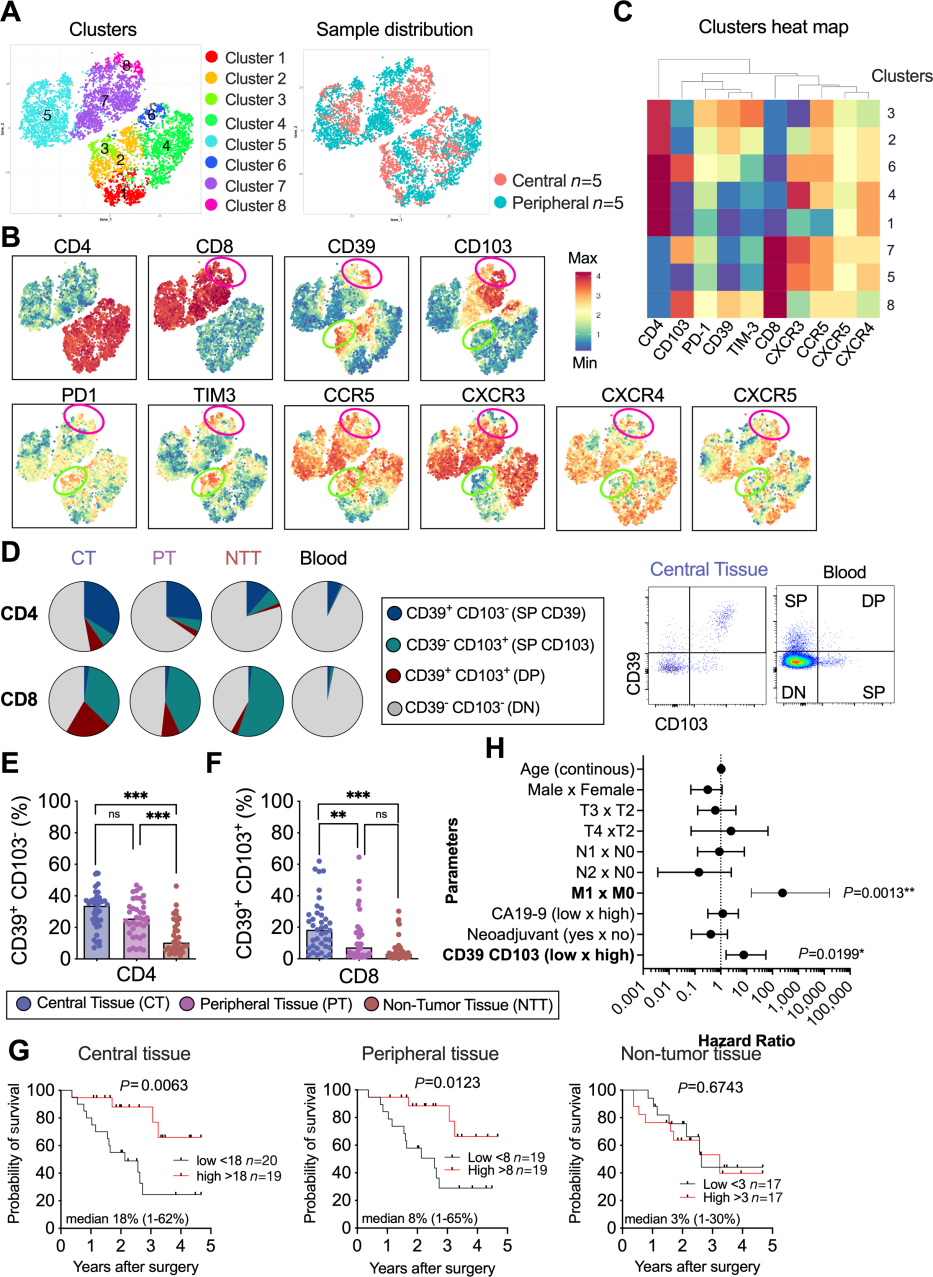
Tumor-infiltrating CD39^+^CD103^+^ T cells. **A,** A t-SNE analysis showing cluster point plots (1–8) and sample distribution in central (*n* = 5) and peripheral (*n* = 5) tumor tissues on 10-parameters flow cytometry analyses. **B,** Distribution of each parameter as a single cell level. The color shows the median fluorescence expression (MFI) of each marker. Green circles highlight cluster 3 representing CD4^+^ CD39^+^ T cells. Pink circles highlight cluster 8 representing CD8^+^ CD39^+^ CD103^+^ T cells. **C,** Heat map showing the distribution of each parameter for every cluster (1–8). The color shows the MFI of each marker (red: high MFI; blue: low MFI). **D,** Left, Pie charts showing the median values of CD39 SP, DP, CD103 SP, and DN CD4^+^ and CD8^+^ T cells in central tumor tissues (CT) *n* = 39, peripheral tumor tissues (PT) *n* = 38, non-tumor tissues (NTT) *n* = 34 and patient PBMCs (blood; *n* = 38; right). Flow cytometry dot plots showing the gating strategy to identify CD39^+^ CD103^−^ (CD39 SP), CD39^+^ CD103^+^ (DP), CD39^−^ CD103^+^ (CD103 SP), and CD39^−^ CD103^−^ (DN) on CD8^+^ T cells. Proportion of CD4^+^ T cells SP CD39 (CT *n* = 39, PF *n* = 38, NTT *n* = 34; **E**) and DP CD8^+^ T cells (**F**) in CT *n* = 39 and PF *n* = 38, NTT *n* = 34. **G,** Kaplan–Meier survival curves were performed on patients with high and low frequency of DP CD8^+^ T cells out of CD8^+^ T cells in central, peripheral, and non-tumor tissues. log-rank test was performed to detect statistical significance. **H,** Forest plots representing survival HRs of the clinicopathologic parameters and DP CD8^+^ T cells in central pancreatic tumor tissues defined in multivariate Cox proportional hazards regression analysis. **E** and **F,** Friedman test followed by Dunn test was used to evaluate significant difference between groups. *, *P* < 0.05; **, *P* < 0.01; ***, *P* < 0.001; ns, not significant. T2, Tumor >2 cm; T3, Tumor > 4 cm; T4, Tumor involves coeliac axis, superior mesenteric artery and/or common hepatic artery; N0, No regional lymph node metastasis; N1, Metastases in one to three regional lymph nodes; N2, Metastases in four or more regional lymph nodes; M0, No distant metastasis; M1, Distant metastasis.

Previous studies have shown that CD4^+^ CD39^+^ T cells (28) and CD8^+^ T cells double positive (DP) for CD39 and CD103 contain a high frequency of tumor-reactive T cells (26), but these subsets of T cells have not yet been studied in pancreatic cancer. On the basis of 11 different markers, t-SNE analysis of five central tissues and five paired peripheral tissues revealed eight different clusters (1–8) of T cells present in both compartments (Fig. 4A–C). As shown in the t-SNE dot plots (Fig. 4B) and heat map (Fig. 4C), cluster 3 represented CD4^+^ CD39^+^ T cells (highlighted in green circles), whereas cluster 8 represented DP CD8^+^ T cells (highlighted in pink circles). In line with previous studies of other types of solid tumors, the t-SNE analysis and heat map showed that CD4^+^ CD39^+^ T cells and DP CD8^+^ T cells expressed high levels of PD-1 and TIM-3 (Fig. 4B and C). Moreover, the heat map also revealed that CD4^+^ CD39^+^ T cells and DP CD8^+^ T cells expressed less CXCR3 and CXCR4 than the other CD4^+^ and CD8^+^ T-cell clusters (Fig. 4C).

Next, we identified different subpopulations of CD4^+^ and CD8^+^ T cells in the central, peripheral, and adjacent non-tumor tissues based on CD39 and CD103 expression by manual gating (Fig. 4D). The majority of CD4^+^ T cells presented a double negative (DN) phenotype for CD39 and CD103 in all three tissue compartments. A substantial proportion of CD4^+^ T cells from central (median 34%) and peripheral (median 27%) tissues were single positive (SP) for CD39, and this population was significantly lower in non-tumor tissues (median 11%; Fig. 4E). A small proportion of CD4^+^ T cells were DP, and they were more dominant in central and peripheral tissues than in non-tumor tissues (Fig. 4D). It is well established that regulatory T cells (Treg) are prevalent in pancreatic cancer (35) and that they express high levels of CD39 (36). Therefore, we assessed whether CD39^+^ CD4^+^ T cells also express FOXP3 and CD25 as phenotypic markers of Tregs. Supplementary Figure S4 shows that approximately 40% of CD4^+^ T cells in central tissues were Tregs, and that the majority of CD39^+^ CD4^+^ T cells were also positive for CD25 and FOXP3. This suggests that most CD39^+^ CD4^+^ T cells display a Treg phenotype and may, therefore, not function as tumor-reactive T cells in pancreatic cancer.

The majority of CD8^+^ T cells in tissues were either DN for CD39 and CD103, or SP for CD103 (Fig. 4D). However, we also identified a population of DP CD8^+^ T cells that was mainly found in central tissues (median 21%) compared with peripheral (median 9%) and non-tumor tissues (median 3%; Fig. 4D and F), suggesting an accumulation of these T cells in tumor rich areas. patient PBMCs contained a median of 6% of CD4^+^ CD39 SP T cells and 0% of CD8^+^ DP T cells (Fig. 4D).

### CD39^+^CD103^+^ CD8^+^ T Cells are Associated to Better OS

We next analyzed the impact of the frequency of DP CD8^+^ T cells out of CD8^+^ T cells in central, peripheral, and non-tumor tissues on patient outcome. We found that a high frequency of DP CD8^+^ T cells in central and peripheral tissues, but not in non-tumor tissues, was associated with better prognosis (Fig. 4G). The total number of DP CD8^+^ T cells/mg of tissue was also associated with a better prognosis in central tissues (Supplementary Fig. S5A). To further estimate the strength of DP CD8^+^ T cells in predicting pancreatic cancer patient survival, we combined the presence of DP CD8^+^ T cells in central tissues with other clinicopathologic factors including age, sex, T, N, M stage, levels of CA19-9, and neoadjuvant chemotherapy in a multivariate Cox proportional hazards regression analysis. Only the M stage and proportion of DP CD8^+^ T cells were significant for OS (Fig. 4H). In contrast, neither SP CD39 nor DP CD4^+^ T cells from CD4^+^ T cells were associated with patient prognosis (Supplementary Fig. S5B).

### CD39 and CD103 T Cells in Pancreatic Tumor Tissues Display an Exhausted Phenotype with a Unique Chemokine Profile

**FIGURE 5:**
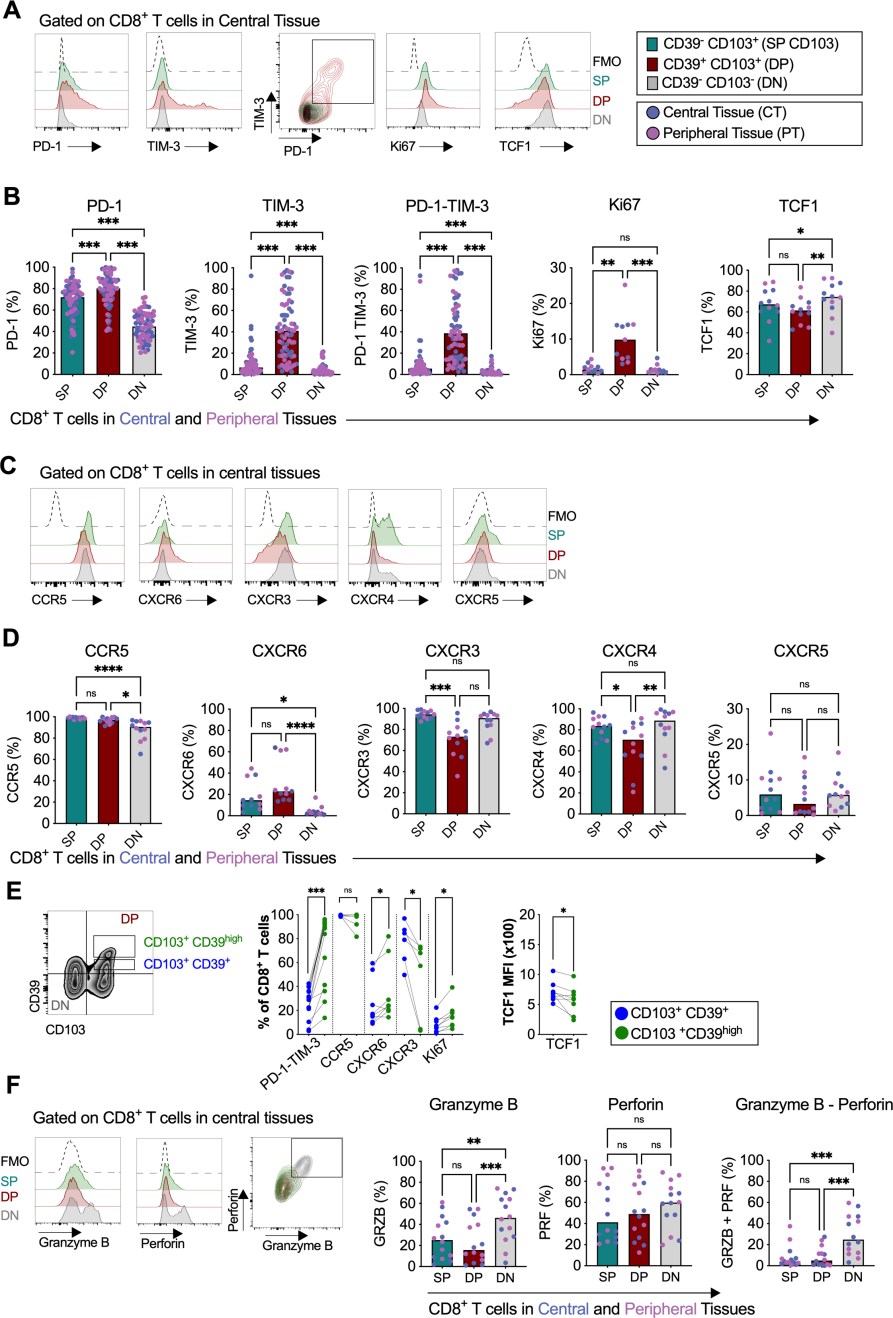
Characterization of CD39^+^CD103^+^ CD8^+^ T cells. **A,** Representative flow cytometry histograms showing the data in B. **B,** Proportion of CD39^−^ CD103^+^ (SP CD103), CD39^+^ CD103^+^ (DP), and CD39^−^ CD103^−^ (DN) CD8^+^ T cells expressing PD-1 (*n* = 36), TIM-3 (*n* = 36), PD-1-TIM-3 (*n* = 36), Ki67 (*n* = 6), and TCF1 (*n* = 6) in central and peripheral tissues. **C,** Representative flow cytometry histograms showing the data in D. **D,** Proportion of DP and DN CD8^+^ T cells expressing CCR5, CXCR6, CXCR3, CXCR4, and CXCR5 in central and peripheral tissues of 6 patients. **E,** Representative zebra plots showing the gating strategy for DP CD8^+^ T cells with high expression of CD39 (CD103^+^ CD39^high^) and low expression of CD39 (CD103^+^ CD39^+^). **F,** Proportion of CD103^+^ CD39^+^ and CD103^+^ CD39^high^ expressing PD-1-TIM-3, CCR5, CXCR3, CXCR6, Ki67, and TCF1. **F,** Left, Representative flow cytometry histograms. Right, Proportion of granzyme B, perforin, and granzyme B-perforin on CD39^+^ CD103^+^ DP and CD39^−^ CD103^−^ DN CD8^+^ T cells from central and peripheral tumor tissues from 7 patients. **D** and **F,** Friedman test followed by Dunn test was used to evaluate significant difference between groups. *, *P* < 0.05; **, *P* < 0.01; ***, *P* < 0.001; ns, not significant. **E,** Wilcoxon matched pairs signed-rank test was used to detect statistically significant differences. *, *P* < 0.05; **, *P* < 0.01; ***, *P* < 0.001; ns, not significant.

Next, we investigated the phenotype of this T-cell subset in pancreatic cancer. In line with studies on other types of solid tumors (26, 27), DP CD8^+^ T cells in central tissues expressed higher levels of PD-1 and TIM-3 and coexpressed more PD-1 and TIM-3 than CD103 SP and DN CD8^+^ T cells (Fig. 5A and B). DP CD8^+^ T cells in central and peripheral tissues also presented higher frequencies of Ki67 expression, suggesting proliferation due to recent encounters with cognate antigens in the tumor microenvironment (Fig. 5A and B). The transcription factor TCF1 is associated with an effector phenotype in CD8^+^ T cells (37). TCF1 was expressed by DP CD8^+^ T cells but was expressed at lower levels compared with DN CD8^+^ T cells (Fig. 5A and B). Together, these data suggest that there is an accumulation of putative tumor-specific T cells in the central and peripheral PDAC tissues, which display an effector but exhausted phenotype.

Similarly, CD39 CD4^+^ SP T cells and DP CD4^+^ T cells in the central and peripheral tissues expressed more PD-1, TIM-3, and PD-1 and TIM-3 coexpression than DN CD4^+^ T cells (Supplementary Fig. S6A and S6B). CD39^+^ CD4^+^ T cells also expressed higher frequencies of Ki67 and lower levels of TCF1 than DN CD4^+^ T cells did (Supplementary Fig. S6A and S6B).

Analysis of chemokine receptor expression showed that DP CD8^+^ T cells to a larger degree expressed CCR5 and CXCR6 and, to a lesser extent CXCR3, and CXCR4, compared with DN CD8^+^ T cells (Fig. 5C and D). No significant differences were found in the expression of CXCR5 between DP and DN CD8^+^ T cells (Fig. 5C and D). Chemokine receptor expression on CD39^+^ CD4^+^ T cells showed a similar pattern to that of DP CD8^+^ T cells (Supplementary Fig. S6C).

Interestingly, as shown in the representative zebra plots (Fig. 5E), DP CD8^+^ T cells from some patients contained a population with higher intensity expression of CD39. DP CD39^high^ CD8^+^ T cells showed increased expression of PD-1, TIM-3, and CXCR6, and a higher proliferative capacity, but a lower expression of CXCR3 and TCF1 than DP CD39^+^ CD8^+^ T cells (Fig. 5E). This suggests that DP CD39^high^ CD8^+^ T cells to a larger extent encounter their cognate antigens in the tumor microenvironment and acquire an exhausted phenotype.

Altogether, putatively tumor-reactive DP CD8^+^ T cells expressed higher levels of coinhibitory markers, had a higher proliferative capacity, and acquired a unique chemokine receptor expression profile by the upregulation of CCR5 and CXCR6 and downregulation of CXCR3 and CXCR4. Moreover, this phenotype was more pronounced in DP CD8^+^ T cells with a higher intensity expression of CD39.

### DP CD8^+^ T Cells Present a Functionally Exhausted Phenotype

Next, we assessed the cytotoxic potential of DP CD8^+^ and CD39^+^ CD4^+^ T cells by measuring the basal level of expression of the cytolytic molecules, granzyme B and perforin. The putatively tumor-reactive DP CD8^+^ T cells presented lower levels of granzyme B and coexpressed less granzyme B and perforin than DN and SP CD8^+^ T cells (Fig. 5F). CD39^+^ CD4^+^ T cells also displayed a more functionally exhausted phenotype than DN CD4^+^ T cells with lower levels of granzyme B (Supplementary Fig. S6D).

### Presurgical Chemotherapy Instigates Alterations in the Immune Landscape

Only about 15% of patients undergo upfront surgery upon diagnosis (38). For patients with borderline resectable disease, neoadjuvant chemotherapy (Neo-CT) is an option to potentially downstage their condition and make the tumors resectable (38). However, there are yet few studies analyzing the changes in the immune landscape in response to therapy.

**FIGURE 6:**
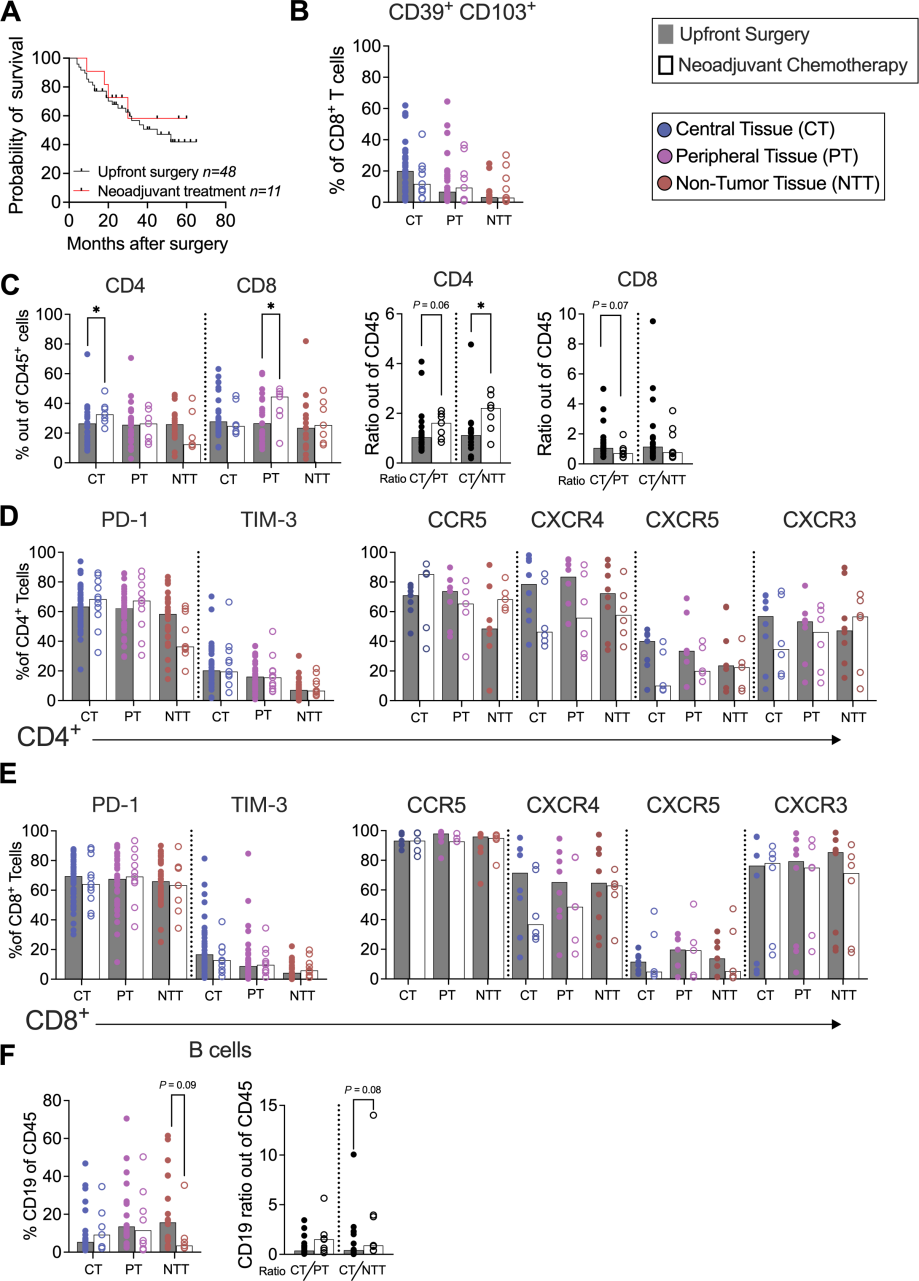
Effects of presurgical chemotherapy on the immune landscape. The immune landscape for patients that underwent upfront surgery (gray squares) was compared with the patients that underwent neoadjuvant chemotherapy followed by surgery (white squares). **A,** Kaplan–Meier survival curves comparing the two groups. **B,** Proportion of CD39^+^ CD103^+^ CD8^+^ T cells. **C,** Left, Proportion of CD4^+^ and CD8^+^ T cells out of CD45^+^ cells. Right, Ratio of cells in central tissues relative to peripheral and non-tumor tissues for CD4^+^ and CD8^+^ T cells out of CD45^+^ cells. Proportion of CD4^+^ T cells (**D**) and CD8^+^ T cells (**E**) expressing PD-1, TIM-3, CCR5, CXCR4, CXCR5, and CXCR3. **F,** Left, Proportion of CD19^+^ cells out of CD45^+^ cells. Right, Proportion of CD19 cells in central tissues relative to peripheral and non-tumor tissues. Mann–Whitney U test was used to detect statistically significant differences. *, *P* < 0.05; **, *P* < 0.01; ***, *P* < 0.001; ns, not significant.

In the current cohort, 19% of patients underwent Neo-CT. Thus, we sought to compare the immune landscape of these patients with those who underwent upfront surgery. In our cohort, Neo-CT followed by surgery did not demonstrate a survival advantage compared with upfront surgery (Fig. 6A). There was no significant difference in infiltration of DP CD8^+^ T cells into the central or peripheral tumor-rich tissues (Fig. 6B), suggesting that Neo-CT does not affect tumor infiltration of putatively tumor-reactive T cells.

Interestingly, patients undergoing Neo-CT exhibited a higher proportion of CD4^+^ T cells within central tissues, whereas CD8^+^ T cells were higher within peripheral tissues compared with those who underwent upfront surgery (Fig. 6C). Further analysis into the spatial distribution of CD4^+^ and CD8^+^ T cells showed that the relative distribution of CD4^+^ T cells was increased in central compared with peripheral and non-tumor tissues in patients receiving Neo-CT compared with upfront surgery. In contrast, there was a trend for a reduced allocation of CD8^+^ T cells in central compared with peripheral tumor tissues in patients who underwent Neo-CT (Fig. 6C). This may suggest that Neo-CT promotes an increased migration of CD4^+^ T cells toward tumor-rich areas, whereas CD8^+^ T cells accumulates in peritumoral areas. There was no significant differences in the expression of chemokine receptor on T cells between the groups that possibly could explain this phenomenon (Fig. 6D and E), but the analysis was based on a limited number of samples. The median proportion of T cells expressing CXCR4 was lower in the Neo-CT–treated group, which potentially might confer a better migratory capacity of T cells within the tumor microenvironment. The expression of coinhibitory markers did not appear to be affected by Neo-CT (Fig. 6D and E). Finally, analysis of the relative distribution of B cells across the tissues showed a trend of higher tumor infiltration in Neo-CT–treated patients compared with upfront surgery (Fig. 6F).

## Discussion

Identifying novel treatments to overcome the unique biology of pancreatic cancer is imperative to reduce the high mortality rate of this disease. Probably because of the low mutational burden, poor T-cell infiltration, and predominant immunosuppressive tumor stroma, current immunotherapeutic strategies fail to yield any response. To understand this failure, more knowledge of the location, phenotype, and functional characteristics of infiltrating T cells is crucial. Because the infiltration of T cells in pancreatic cancer has been associated with improved survival (2–4), there is a rationale to find antigen-specific T cells for emerging T-cell transfer therapies. Indeed, 8 out of 16 patients who received individualized mRNA neoantigen vaccines in a recent phase I clinical trial had a strong and durable T-cell response against neoantigens (39).

To provide a more complete picture of the localization and phenotype of T cells in pancreatic cancer, we characterized T cells in three different tissue compartments: the central tissue (rich in tumor cells), peripheral tissue (containing fewer tumor cells and more parenchymal remnants), and adjacent non-tumor tissue (containing no tumor cells, but in some donors, it could contain some reactive stroma). We found that T cells are present in the three tissue compartments, and unlike other studies on pancreatic cancer (2–4), a high number of CD8^+^ T cells did not correlate with a better prognosis. This supports the notion of infiltration of tumor-nonspecific bystander T cells (40). However, in line with previous studies tumor-infiltrating T cells presented high expression of coinhibitory markers (29, 30). Because of the small tissue size and low number of infiltrating T cells, we could not perform T-cell reactivity analysis or next-generation sequencing analysis to distinguish antigen-specific T cells. However, recent studies in colon, ovarian, lung, endometrial, and head and neck cancers have linked a subset of clonally expanded, tumor-reactive T cells with the coexpression of CD39 and CD103 (26, 27, 41, 42).

In the current study, we identified a population of CD39 and CD103 DP CD8^+^ T cells in pancreatic cancer tissues, confirming findings from a recent study (30). However, in the current study, we bring additional insights into the relevance and location of CD39^+^CD103^+^CD8^+^ T cells in pancreatic cancer. DP CD8^+^ T cells were enriched in central tissues compared with peripheral and non-tumor tissues, and a high presence of DP CD8^+^ T cells in tumor-rich tissues, but not in non-tumor tissues, was correlated with better OS. This underscores the importance of this T-cell subset in pancreatic cancer and its potential physical interaction with tumor cells. Consistent with a recent encounter with tumor antigens, DP CD8^+^ T cells expressed higher frequencies of the proliferative marker Ki67, expressed more coinhibitory markers (PD-1 and TIM-3), and presented a less cytotoxic phenotype with lower basal levels of granzyme B and perforin than DN CD8^+^ T cells. These traits were even more pronounced in DP CD8^+^ T cells with a higher expression of CD39. Previous studies have also shown that DP CD8^+^ T cells express high levels of coinhibitory receptors, are proliferative (27, 42), and that upon stimulation, DP CD8^+^ T cells released less IFNγ and TNFα (26). However, contrary to our data, these studies showed higher expression of granzyme B in DP CD8^+^ T cells (26, 27). We could argue that we showed protein levels in unstimulated cells, while Laumont and colleagues showed gene expression (27), which might not always correlate with protein levels (43). Duhen and colleagues investigated granzyme B levels in phorbol 12-myristate 13-acetate/ionomycin-stimulated cells (26). Owing to the low number of T cells, we could not isolate DP CD8^+^ T cells to investigate their cytotoxicity upon stimulation.

The expression patterns of different chemokine receptors can affect the capacity of T cells to migrate toward malignant cells. Interestingly, whereas CCR5, CXCR3, CXCR4, CXCR5, and CXCR6 were uniformly expressed on CD4^+^ and CD8^+^ T cells across the three tissue compartments, DP CD8^+^ T cells presented a unique chemokine profile with higher expression levels of CCR5 and CXCR6, but lower levels of CXCR3 and CXCR4 compared with DN CD8^+^ T cells. The expression of CXCR3 and CXCR4 has been shown to be downregulated upon activation and antigen exposure (44, 45), which supports the notion that DP CD8^+^ T cells with lower expression of CXCR3 and CXCR4 encounter their cognate tumor antigen. The downregulation of CXCR4 might also enhance the infiltration of DP T cells into tumor-rich areas, because pancreatic cancer–associated fibroblasts can retain T cells in the tumor stroma through the CXCR4-CXCL12 axis (18). CXCR6 is highly expressed in tumor-specific CD8^+^ T cells and enhances CD8^+^ T-cell retention in tumor nests, which is crucial for persistent and effective immunosurveillance (14, 15). Here, we showed that CXCR6 was expressed nine times more in DP CD8^+^ T cells than in DN CD8^+^ T cells, which could indicate a relevant function of this chemokine receptor in pancreatic cancer immunosurveillance. A study in melanoma showed that tumor-reactive cytotoxic T cells lose the expression of the stem-like transcription factor TCF1 and highly upregulate the expression of chemokine receptors CXCR6 and CCR5 but downregulate CXCR3 (46). The same study also showed that CXCR6 promotes the migration and interaction of tumor-reactive T cells with intratumoral dendritic cells and subsequent antitumor activity (46). Altogether, these results provide further evidence that DP CD8^+^ T cells, with their unique chemokine profile, can identify tumor antigen-specific T cells in pancreatic cancer. In addition to CD39 and CD103, other extracellular markers such as CXCR6 could be used to isolate antigen-specific T cells and develop T cell–based immunotherapies. However, further studies on the functionality of CXCR6 in pancreatic cancer tissues are required.

Whether DP CD8^+^ T cells with low basal expression of the cytotoxic granzyme B molecule, low expression of TCF1, and high expression of PD-1 and TIM-3 have acquired a fully dysfunctional phenotype or whether they would respond to immune checkpoint inhibitors remains to be clarified. However, a previous study on colorectal cancer successfully showed the reactivity of isolated and expanded DP CD8^+^ T cells against neoantigens, proving their cytotoxic functionality (41). It has been suggested that the expression of CX3CR1 by cytotoxic TCF1^neg^ PD-1^+^ TIM-3^+^ CD8^+^ T cells identifies proliferative and functional T cells, whereas the expression of CD101 in cytotoxic TCF1^neg^ PD-1^+^ TIM-3^+^ CD8^+^ T cells identifies fully exhausted and dysfunctional T cells during chronic viral infection (47). Owing to the limited number of samples, we could not explore these markers in the DP CD8^+^ T-cell population, leaving this question open to future investigations. However, a study using a melanoma mouse model showed that combined blockade of CD39 and TIM-3 reduced tumor growth and increased survival by reactivating dysfunctional CD39^+^ TIM-3^+^ CD8^+^ TILs (48). Another *in vitro* study in pancreatic cancer showed that dual blockade of PD-1 and TIGIT in TILs also rescued T cell effector function (30). These studies suggest that targeting immune checkpoint inhibitors together with other novel markers could reactivate exhausted T cells and enhance the efficacy of T-cell therapies.

We have also shown a prevalent infiltration of CD39^+^ CD4^+^ T cells in central and peripheral tissues compared with non-tumor tissues. This subset has been described as tumor antigen-specific T cells in other types of cancers (28). However, in line with other studies on pancreatic cancer (35), most CD39^+^ CD4^+^ T cells were FOXP3^+^ CD25^+^ Tregs and were not correlated with better survival. We further showed that a high proportion of CD4^+^ T cells express ICOS, which could also be associated with activation and IL10-secreting Tregs (49, 50). Thus, identification of extracellular markers to identify tumor-reactive CD4^+^ T cells in pancreatic cancer warrants further investigation.

The presence of TLS in PDAC has been described and correlated with lower infiltration of immunosuppressive cells and better OS (34, 51). Previous studies investigating chronic viral infections have shown that a subset of CD8^+^ T cells in TLS express CXCR5 with increased expression of PD-1 (16, 52), and that CXCR5 is decisive for T cells to enter B-cell follicles (16, 17). We also found that the median proportion of CXCR5^+^ CD8^+^ T cells in peripheral tissues was higher than that in central tumor tissues, and that CXCR5^+^ cells had higher levels of PD-1 than CXCR5^−^ T cells. Thus, it can be speculated that CXCR5^+^ PD-1^+^ CD8^+^ T cells are found in the TLS, but this warrants further investigation using IHC analyses. The contribution of DP CD8^+^ T cells to TLS formation in pancreatic cancer is unknown. However, in endometrial cancer, tumor-reactive CD39^+^ PD-1^+^ CD8^+^ T cells are a major source of CXCL13 (42), which is a key chemoattractant for B cells and a key component for the formation of TLS (17).

Accumulating data and randomized trials are demonstrating the benefits of neoadjuvant therapy for both borderline resectable and resectable disease (53, 54). This approach not only expands the number of patients eligible for surgery, but also controls potential micrometastasis (53). However, there are yet few studies that have analyzed the shifts in the PDAC tumor microenvironment in response to presurgical therapy, and the existing findings are not conclusive (10, 55, 56). Previous studies have identified changes in cellular composition associated to Neo-CT treatment, inducing tumor progression characteristics and the infiltration of tumor-promoting monocytes (55). In contrast, others have shown an increase in the infiltration of antitumor immune cells (56). We found that Neo-CT–treated patients displayed an elevated infiltration of CD4^+^ T cells into the central tumor tissues and a tendency for a more dominate peritumoral localization of CD8^+^ T cells. This could suggest a potential impact of chemotherapy on the immune landscape of pancreatic cancer associated to reduced cytotoxicity. However, drawing any solid conclusion would require a larger sample size and matched patients with homogenous treatments.

A limitation of this study is that we did not have complete datasets for all patients. The tissues received from the resected tumors were small, limiting the number of parameters that could be analyzed. The flow cytometry panels were adapted over time to meet new knowledge in the field. The small tissue samples also made it difficult to isolate and study the antigen-specificity of CD8^+^ DP T cells, but this subset was phenotypically very similar to the previously described DP subset with tumor reactivity in other types of solid tumors.

To summarize, our data show that T cells infiltrate both tumor-rich and tumor-free pancreatic tissues, and that PD-1 and several other markers, including chemokine receptors, are expressed uniformly across the tissues. However, central tumor tissues contained significantly higher proportions of TIM-3^+^ and CD39^+^CD103^+^ T cells, indicating that these markers are associated with tumor antigen encounters. DP T cells also displayed a more exhausted phenotype and a different chemokine receptor expression profile than DN T cells, further supporting the fact that they have encountered their cognate antigens and likely represent tumor-specific T cells. Importantly, a high proportion of DP CD8^+^ T cells is associated with increased patient survival. These findings suggest that DP CD8^+^ T cells with a phenotype reminiscent of that of tumor-reactive T cells are present in pancreatic tumors. The abundance of DP CD8^+^ T cells could potentially aid in the selection of appropriate patients for immunotherapy trials for pancreatic cancer.

## Supplementary Material

Supplementary Figure S1 Proportion of tumor/stroma area and immune cells in pancreatic tissues.

Supplementary Figure S2 Overall survival curves for T cells, MAIT cells and B cells.

Supplementary Figure S3 Proportion of T cells co-expressing PD-1 and LAG-3.

Supplementary Figure S4 T regulatory cells out of CD4+ and CD39+ CD4+ T cells.

Supplementary Figure S5 Overall survival curves for DP CD8 T cells and SPCD39, DP CD4 T cells.

Supplementary Figure S6 Characterization of CD39+CD4+ T cells

Supplementary Table S1 Antibodies used in flow cytometry.
