# Supplementary material for: Exhausted Tumor-infiltrating CD39+CD103+ CD8+ T Cells Unveil Potential for Increased Survival in Human Pancreatic Cancer
Source: Cancer Res Commun. 2024 Feb 19;4(2):460–74. doi: 10.1158/2767-9764.CRC-23-0405 (PMC10875982; doi:10.1158/2767-9764.CRC-23-0405)
Supplement: Supplementary Figure S1 — Proportion of tumor/stroma area and immune cells in pancreatic tissues. [file crc-23-0405-s01.docx]

**Supplementary Figure S1**

**A**


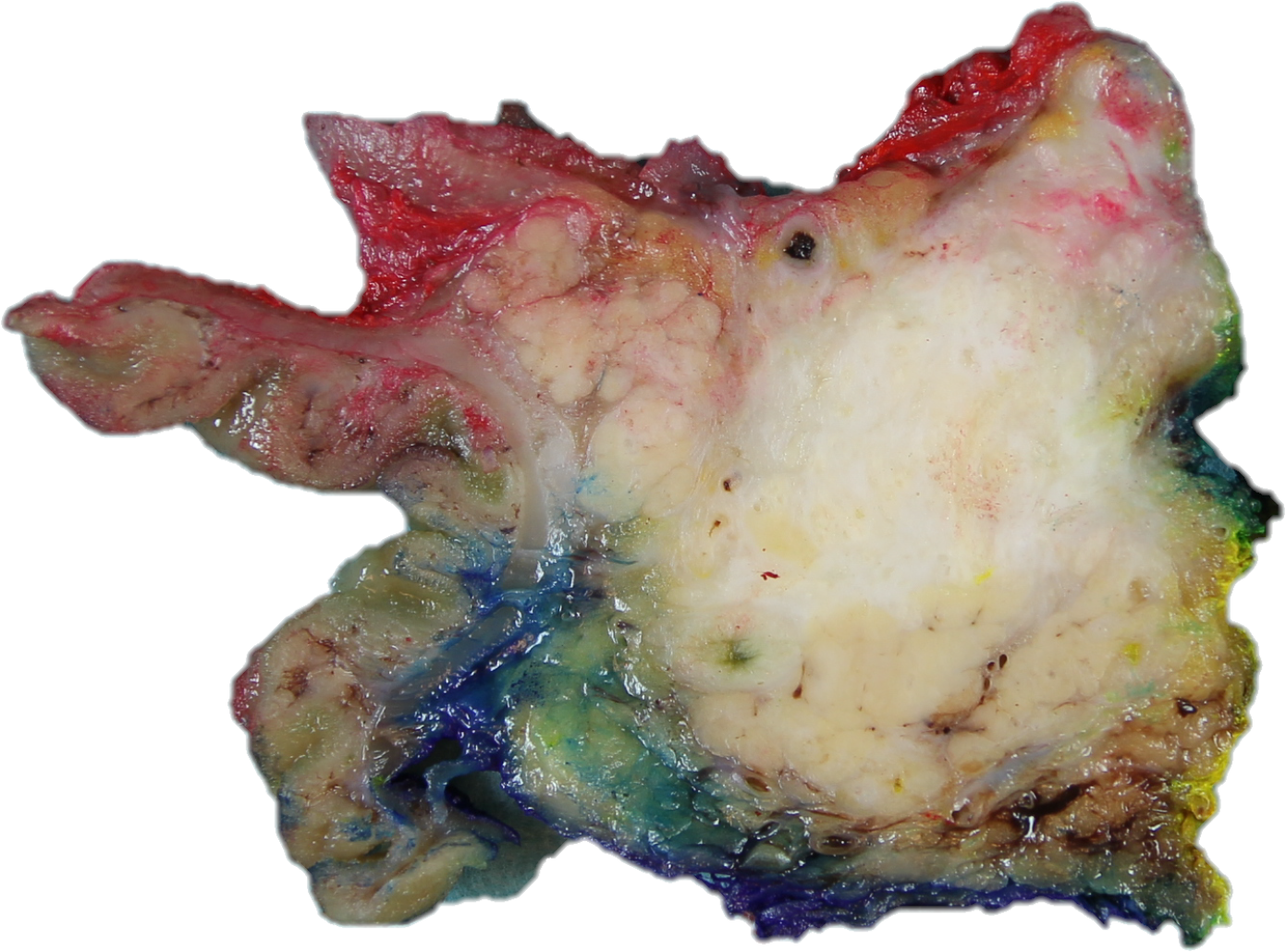


**
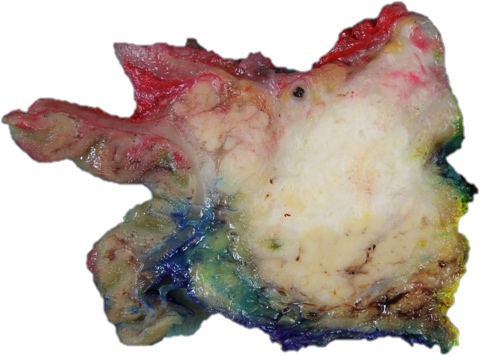
**

**B**


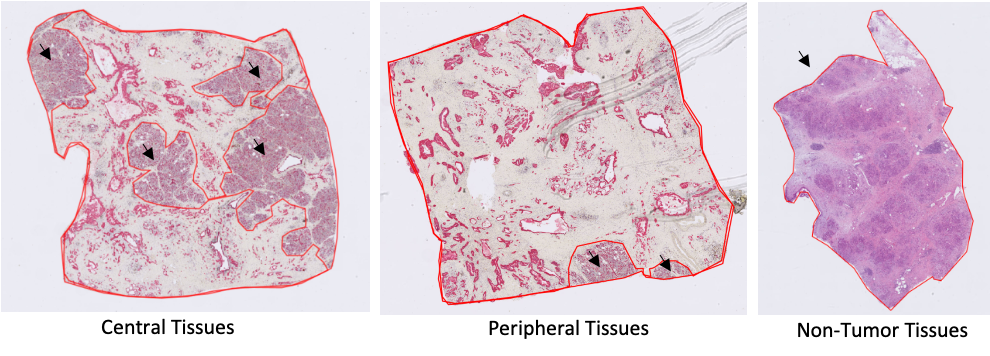

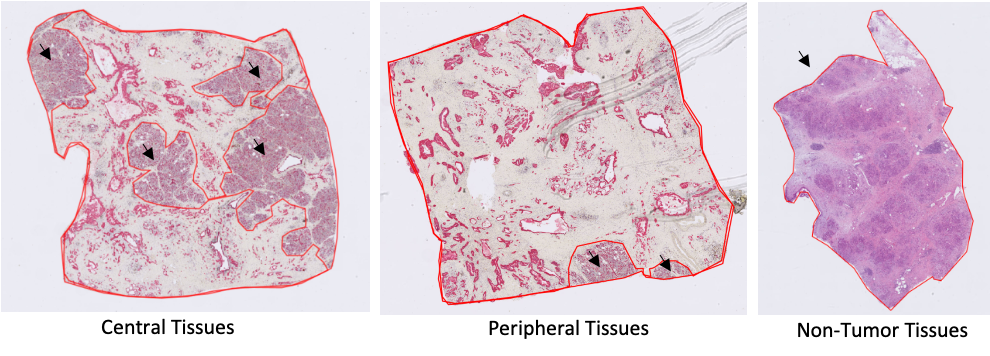

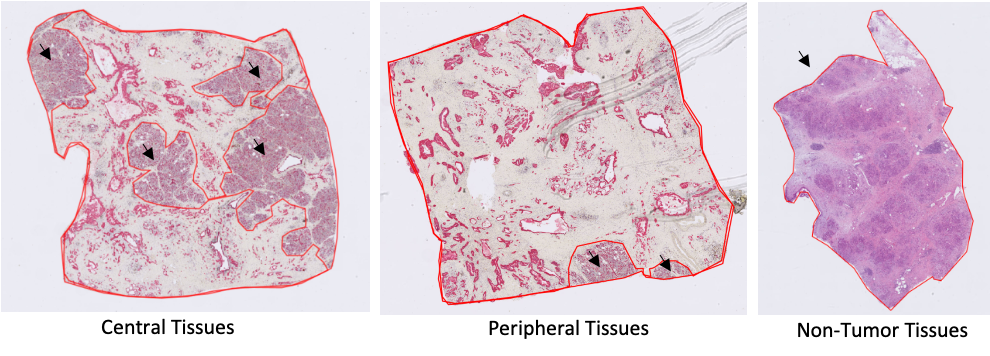


Central Tissue

Peripheral Tissue

Non-Tumor Tissue

**C**

**Supplementary Figure S1. Proportion of tumor/stroma area and immune cells in pancreatic tissues. (A)** Sampling technique. A specialized pancreatic pathologist obtained a fresh tissue sample of the central part of the tumor (blue rectangle), the peripheral part of the tumor (red rectangle) and the non-tumorous pancreatic parenchyma (green rectangle). The macro photo illustrates the location of the three tissue samples in an axial tissue section through the head of the pancreas and the adjacent duodenum (orange star) after formalin fixation. The anatomical surfaces of the resection specimen are inked as follows: red – anterior (peritoneal) surface, green – groove of the superior mesenteric vein (SMV), yellow – resection margin in the processus uncinatus facing the superior mesenteric artery (SMA), and blue – posterior (retroperitoneal) surface. Yellow dotted line delineates the tumor (PDAC) lesion in the head of the pancreas on gross visual assessment. **(B)** Annotations were manually annotated in QuPath to quantify the tumor and stroma area in central, peripheral, and non-tumor tissues. Arrows point to areas of normal residual tissue that was excluded from the total area when calculating the proportion of desmoplastic stroma including tumor. **(C)** Representative flow cytometry plots showing the gating strategies to identify CD4^+^ and CD8^+^ T cells from CD3^+^ T cells. The memory phenotype; terminally differentiated ((TD) CD45RA^+^ CCR7^-^)), naïve ((N) CD45RA^+^ CCR7^+^)), central memory ((CM) CD45RA^-^ CCR7^+^)) and effector memory ((EM) CD45RA^-^ CCR7)) Phenotype from CD4^+^ and CD8^+^ T cells. CD19^+^ B cells from CD45^+^ leukocytes and MAIT cells (MR1 tetramer^+^ and CD161^+^) from CD4^+^, CD8^+^ and double negative CD4^-^ and CD8^-^ T cells.
