# Supplementary material for: Exhausted Tumor-infiltrating CD39+CD103+ CD8+ T Cells Unveil Potential for Increased Survival in Human Pancreatic Cancer
Source: Cancer Res Commun. 2024 Feb 19;4(2):460–74. doi: 10.1158/2767-9764.CRC-23-0405 (PMC10875982; doi:10.1158/2767-9764.CRC-23-0405)
Supplement: Supplementary Figure S2 — Overall survival curves for T cells, MAIT cells and B cells. [file crc-23-0405-s02.docx]

**Supplementary Figure S2**

**Supplementary Figure S2. Overall survival curves.** Kaplan-Meier survival curves were performed on patients with high and low frequencies base on the median for: **(A)** Total number of CD4^+^ and CD8^+^ T cells/mg of tissues. **(B)** MAIT cells of CD3^+^ T cells and **(C)** B cells of CD45^+^ leukocytes. Long rank test was performed to detect statistical significance.
