# Supplementary material for: Exhausted Tumor-infiltrating CD39+CD103+ CD8+ T Cells Unveil Potential for Increased Survival in Human Pancreatic Cancer
Source: Cancer Res Commun. 2024 Feb 19;4(2):460–74. doi: 10.1158/2767-9764.CRC-23-0405 (PMC10875982; doi:10.1158/2767-9764.CRC-23-0405)
Supplement: Supplementary Figure S3 — Proportion of T cells co-expressing PD-1 and LAG-3. [file crc-23-0405-s03.docx]

**Supplementary Figure S3**

**
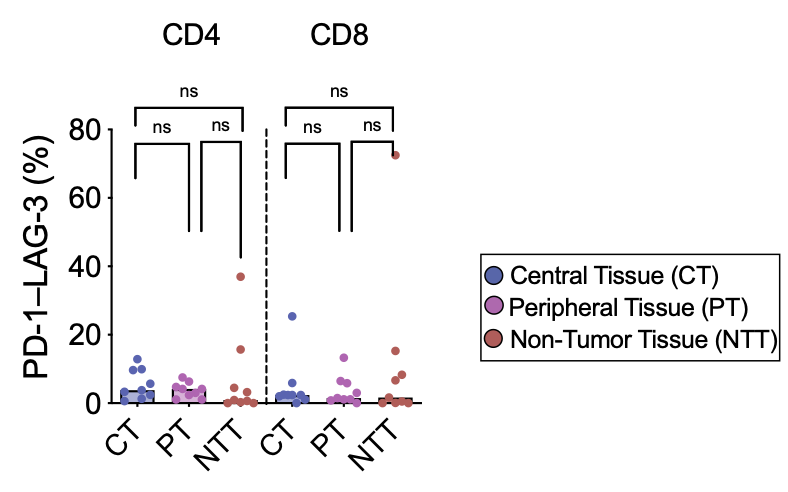
**

**Supplementary Figure S3.**  Proportion of CD4^+^ and CD8^+^ T cells co-expressing PD-1 and LAG-3 co-inhibitory markers (n=9). Friedman’s test followed by Dunn´s test was used to evaluate significant difference between groups (CT, PT, NTT), ns; not significant.
