# Supplementary material for: Exhausted Tumor-infiltrating CD39+CD103+ CD8+ T Cells Unveil Potential for Increased Survival in Human Pancreatic Cancer
Source: Cancer Res Commun. 2024 Feb 19;4(2):460–74. doi: 10.1158/2767-9764.CRC-23-0405 (PMC10875982; doi:10.1158/2767-9764.CRC-23-0405)
Supplement: Supplementary Figure S4 — T regulatory cells out of CD4+ and CD39+ CD4+ T cells. [file crc-23-0405-s04.docx]

**Supplementary Figure S4**

**Supplementary Figure S4. The majority of CD4^+^ and CD39^+^ CD4^+^ T cells are T regulatory cells.** (left) Proportion of T regulatory cells (Tregs CD25^+^ FOXP3^+^) from CD4^+^ T cells (right) proportion of Tregs from CD39^+^ CD4^+^ T cells (n=3). (bottom) Representative zebra plots.
