# Supplementary material for: Exhausted Tumor-infiltrating CD39+CD103+ CD8+ T Cells Unveil Potential for Increased Survival in Human Pancreatic Cancer
Source: Cancer Res Commun. 2024 Feb 19;4(2):460–74. doi: 10.1158/2767-9764.CRC-23-0405 (PMC10875982; doi:10.1158/2767-9764.CRC-23-0405)
Supplement: Supplementary Figure S5 — Overall survival curves for DP CD8 T cells and SPCD39, DP CD4 T cells. [file crc-23-0405-s05.docx]

**Supplementary Figure S5**

**Supplementary Figure S5. Overall survival curves.** Kaplan-Meier survival curves were performed on patients with high and low frequencies base on the median for: **(A)** Total number of DP CD8^+^ T cells/mg of tissue. **(B)** Proportion of SP CD39 and DP out of CD4^+^. Long rank test was performed to detect statistical significance. **p*<0.05, ***p*<0.01, ****p*<0.001, *p*>0.05 not significant.
