# Supplementary material for: Exhausted Tumor-infiltrating CD39+CD103+ CD8+ T Cells Unveil Potential for Increased Survival in Human Pancreatic Cancer
Source: Cancer Res Commun. 2024 Feb 19;4(2):460–74. doi: 10.1158/2767-9764.CRC-23-0405 (PMC10875982; doi:10.1158/2767-9764.CRC-23-0405)
Supplement: Supplementary Figure S6 — Characterization of CD39+CD4+ T cells [file crc-23-0405-s06.docx]

**Supplementary Figure S6**

**Supplementary Figure S6. Characterization of CD39^+^CD4^+^ T cells.** **(A-B)**. Proportion of CD39^+^ CD103^-^ (SP CD39), CD39^+^ CD103^+^ (DP) and CD39^-^ CD103^-^ (DN) CD4^+^ T cells expressing PD-1 (n=37), TIM-3 (n=37) PD-1-TIM-3 (n=37), Ki67 (n=6), and TCF1 (n=6) in (**A)** central tissues and **(B)** peripheral tissues. **(C)** Proportion of SPCD39 and DN CD4^+^ T cells expressing CCR5, CXCR6, CXCR3, CXCR4, and CXCR5 in central and peripheral tissues of 6 patients**. (D)** Proportion of Granzyme B, Perforin, Granzyme B-Perforin co-expression (n=8) on CD4^+^ CD39^+^ and DN CD4^+^ T cells from central and peripheral tumor tissues (n=6). **(A-B)** Friedman´s test followed by Dunn´s test was used to evaluate significant difference between groups. **(C-D)** Wilcoxon matched pairs signed rank test was used to detect statistically significant differences. *P<0.05, **P<0.01, ***P<0.001, ns; not significant.
