# Supplementary material for: Exhausted Tumor-infiltrating CD39+CD103+ CD8+ T Cells Unveil Potential for Increased Survival in Human Pancreatic Cancer
Source: Cancer Res Commun. 2024 Feb 19;4(2):460–74. doi: 10.1158/2767-9764.CRC-23-0405 (PMC10875982; doi:10.1158/2767-9764.CRC-23-0405)
Supplement: Supplementary Table S1 — Antibodies used in flow cytometry. [file crc-23-0405-s07.docx]

**Supplementary Table S1**

**Supplementary Table S1.** Antibodies used in flow cytometry.

| Markers | Fluorochrome | Clone | Company | Catalogue number | Staining |
| --- | --- | --- | --- | --- | --- |
| CD3 | PE-Cy-7 | UCHT1 | BD | 563423 | EC |
| CD3 | FITC | UCHT1 | BD | 555916 | EC |
| CD3 | V450 | UCHT1 | BD | 560365 | EC |
| CD3 | BV780 | UCHT1 | Biolegend | 300472 | EC |
| CD4 | A700 | RPA-T4 | BD | 557922 | EC |
| CD4 | V500 | RPA-T4 | BD | 560768 | EC |
| CD8 | APC-Cy7 | SK1 | BD | 557834 | EC |
| CD8 | A700 | RPA-T8 | BD | 557945 | EC |
| CD8 | PE-Cy-7 | RPA-T8 | BD | 557746 | EC |
| CD8 | APC | RPA-T8 | BD | 555369 | EC |
| CD8 | PECF594 | RPA-T8 | BD | 562282 | EC |
| CD45 | A700 | H130 | BD | 560566 | EC |
| CD45 | PE-Cy-7 | H130 | BD | 557748 | EC |
| CD69 | PE-Cy-7 | L78 | BD | 335792 | EC |
| CD45RA | PE-Cy7 | HI100 | BD | 560675 | EC |
| CCR7 | PECF594 | 150503 | BD | 562381 | EC |
| CD19 | PECF594 | HIB19 | BD | 562294 | EC |
| CD19 | APC | Mab11 | BD | 34791 | EC |
| CD161 | PE | HP-3G10 | Biolegend | 339904 | EC |
| CD39 | BV421 | TU66 | BD | 563679 | EC |
| CD103 | PE | Ber-ACT8 | BD | 550260 | EC |
| CXCR4 | PE-Cy7 | 12G5 | Biolegend | 306514 | EC |
| CXCR3 | APC | 1C6 | BD | 550967 | EC |
| CXCR5 | PE | J252D4 | Biolegend | 356904 | EC |
| CXCR5 | BV780 | J252D4 | Biolegend | 356936 | EC |
| CCR5 | BV421 | 3A9 | BD | 565000 | EC |
| CCR5 | PECF594 | 2D7 | BD | 562456 | EC |
| CXCR6 | PERCP-Cy5.5 | K041E5 | Biolegend | 356010 | EC |
| CTLA-4 | FITC | A3.4H2.H12 | LS-Bio | LS-C34558-250 | EC |
| TIM-3 | APC | F38-2E2 | Miltenyi | 130098936 | EC |
| TIM-3 | BV650 | F38-2E2 | Biolegend | 340528 | EC |
| PD-1 | BV421 | EH12.1 | BD | 562516 | EC |
| PD-1 | BV510 | EH12.1 | BD | 563076 | EC |
| LAG-3 | PE | REA351 | Miltenyi | 130105452 | EC |
| LAG-3 | APC R700 | T47-530 | BD | 565774 | EC |
| CD25 | PECF594 | M-A251 | BD | 562403 | EC |
| 7AAD | 7AAD | - | BD | 559928 | EC |
| KI67 | PE-Cy7 | B56 | BD | 561283 | IC |
| FOXP-3 | FITC | 236A/E7 | eBioscience | 11477742 | IC |
| Perforin | PECF594 | δG9 | BD | 563763 | IC |
| Granzyme B | FITC | GB11 | BD | 560211 | IC |
| TCF1 | A647 | S33.966 | BD | 566693 | IC |
| Fixable viability Stain | APC-Cy7 | - | BD | 565388 | IC |

Abbreviations; Markers; **CD**, Cluster of differentiation, **MAIT**, Mucosal associated invariant T cells, **CXCR,** Chemokine (C-X-C) motif receptor, **CCR**, Chemokine (C-C) motif receptor, **CTLA-4**, Cytotoxic T lymphocyte-associated antigen 4, **TIM-3**, T-cell immunoglobulin, and mucin-domain containing-3, **PD-1**, Programmed cell death protein-1, **LAG-3**, Lymphocyte activation gene, **FOXP-3**, Forkhead box-3. Companies; **BD**, BD Biosciences (Franklin Lakes, NJ, USA), **BioLegend**, Biolegend (San Diego, CA, USA), **Miltenyi**, Miltenyi Biotec (Bergisch Gladbach, Germany), **eBioscience**, eBioscience, (San Diego, CA, USA). Stainings; **EC**, Extracellular, **IC**, Intracellular.
